# Supplementary material for: Impact of alternative Non-Pharmaceutical Interventions strategies for controlling COVID-19 outbreak in Bangladesh: A modeling study
Source: PLoS One. 2024 Feb 23;19(2):e0293863. doi: 10.1371/journal.pone.0293863 (PMC10890722; doi:10.1371/journal.pone.0293863)
Supplement: S1 File — (DOCX) [file pone.0293863.s001.docx]

**Supplementary (S1): Replication Process and Parameter Values**

The following steps can be followed to download the model used in this study. The CoMo Consortium V17 application can be run using the attached input data sets (templates) to provide results reported in the study.

1. Go to <https://github.com/ocelhay/como/releases/tag/v17.1.1>
2. Expand ‘Asset’
3. Download ‘[CoMo.Setup.17.1.1.exe](https://github.com/ocelhay/como/releases/download/v17.1.1/CoMo.Setup.17.1.1.exe)’ to local PC
4. Open ‘[CoMo.Setup.17.1.1.exe](https://github.com/ocelhay/como/releases/download/v17.1.1/CoMo.Setup.17.1.1.exe)’ to install CoMo Consortium desk-top application.
5. Once installed open ‘CoMo Consortium’
6. In ‘CoMo Consortium’
   1. Select ‘**Visual Calibration**’
   2. Select ‘**Upload Template**’
   3. Select excel file ‘**Template_CoMoCOVID-19App_v17_Bangladesh_May262021v1P_Base.xlsx**’
   4. In CoMo App, select ‘**Calibrate Baseline**’
   5. In CoMo App, select ‘**Validate Baseline**’
   6. In CoMo App, select ‘**Run Scenarios’**
   7. In CoMo App, select ‘**Download Data**’, save file in local PC. Import file into excel. The file ‘COVID19_App_Data’ has daily measures from start and end date of actual/projected period.
   8. In CoMo App, select ‘**Generate Report Based on Current Simulation**’. The MS Word file, ‘CoMo_Model_Report’, has narrative of the results.
   9. Close CoMo Consortium

**Supplementary Table S2: Key Parameter Table with data sources***

| Sheet-Intervention | Parameter | Value | Reference |
| --- | --- | --- | --- |
| Parameters | (changed in v17) Number of exposed people at start date | 10 | B |
| Parameters | (v16.2) Proportion of population with partial immunity at the start date | 32 | B |
| Parameters | Probability of infection given contact (0 to 0.2) | 0.041 | 3, B |
| Parameters | Percentage of all asymptomatic infections that are reported | 1 | B |
| Parameters | Percentage of all symptomatic infections that are reported | 1 | B |
| Country Area Param | Social Contacts Data | Bangladesh | 1 |
| Country Area Param | Mean Household size | 4.1 | Estimated |
| Country Area Param | Mean number of infectious migrants per day | 0 | A |
| Virus Param | Relative infectiousness of incubation phase | 10 | B |
| Virus Param | Average incubation period (1 to 7 days) | 3 | 4-6, B |
| Virus Param | Average duration of symptomatic infection period (1 to 7 days) | 4 | 4, B |
| Virus Param | Probability upon infection of developing clinical symptoms | 25 | 7-9, B |
| Virus Param | Probability upon hospitalisation of requiring ICU admission | 10 | 1 9-12, B |
| Virus Param | Probability upon admission to the ICU of requiring a ventilator | 50 | 15, B |
| Virus Param | Proportion of hospitalised patients needing O2 | 50 | A |
| Hospitalisation Param | Maximum number of hospital surge beds | 130000 | C |
| Hospitalisation Param | Maximum number of ICU beds without ventilators | 2000 | C |
| Hospitalisation Param | Maximum number of ICU beds with ventilators | 2000 | C |
| Hospitalisation Param | Relative percentage of regular daily contacts when hospitalised: | 15 | B |
| Hospitalisation Param | Scaling factor for infection hospitalisation rate: (0.1 to 5) | 1.1 | B |
| Hospitalisation Param | Probability of dying when hospitalised (not req O2): | 15 | 14 |
| Hospitalisation Param | Probability of dying when hospitalised if req O2: | 20 | 14 |
| Self-isolation if Symptomatic | Adherence: | 50 | A |
| (*Self-isolation) Screening | Overdispersion: (1, 2, 3, 4 or 5) | 4 | A |
| (*Self-isolation) Screening | Test Sensitivity: | 80 | A |
| (*Self-isolation) Household Isolation | Days in isolation for average person: | 14 | A |
| (*Self-isolation) Household Isolation | Days to implement maximum quarantine coverage: (1 to 5) | 2 | A |
| (*Self-isolation) Household Isolation | Decrease in the number of other contacts when quarantined: | 20 | A |
| (*Self-isolation) Household Isolation | Increase in the number of contacts at home when quarantined: | 100 | A |
| Social Distancing | Adherence: | 100 | A |
| Handwashing | Efficacy: (0-25%) | 20 | A |
| Mask Wearing | Efficacy: (0-35%) | 15 | A |
| Working at Home | Efficacy: | 85 | A |
| Working at Home | Home contacts inflation due to working from home: | 10 | A |
| School Closures | Home contacts inflation due to school closure: | 20 | A |
| Shielding the Elderly | Efficacy: | 95 | A |
| Shielding the Elderly | Minimum age for elderly shielding: (0 to 100) | 70 | A |
| Vaccination | Time to reach target coverage (1 to 52) | 4 | A |
| Vaccination | (v16.2) Duration of efficacious period | 100 | A |
| Vaccination | (v16.2) Duration of efficacious period if previously infected | 100 | A |
| Vaccination | Efficacy | 100 | A |
| Vaccination | (v16.2) Efficacy if previously infected | 100 | A |
| Mass Testing | Sensitivity | 80 | A |
| Mass Testing | Isolation days | 14 | A |

A: Template_CoMoCOVID-19App_v17.xlsx available at <https://github.com/ocelhay/como/releases/tag/v17.1.1>

B: Template_CoMoCOVID-19App_v17.xlsx, Calibrated

C: DGHS, Bangladesh

References:

1. Prem K, Cook AR, Jit M. Projecting social contact matrices in 152 countries using contact surveys and demographic data. *PLoS Comput Biol* 2017;13(9):e1005697. doi: 10.1371/journal.pcbi.1005697 [published Online First: 2017/09/12]

2. COVID-19: Data 2020 [Available from: <https://www1.nyc.gov/site/doh/covid/covid-19-data.page>.

3. Otto MA. COVID-19 update: Transmission 5% or less among close contacts 2020 [Available from: <https://www.the-hospitalist.org/hospitalist/article/218769/coronavirus-updates/covid-19-update-transmission-5-or-less-among-close>.

4. Linton NM, Kobayashi T, Yang Y, et al. Incubation Period and Other Epidemiological Characteristics of 2019 Novel Coronavirus Infections with Right Truncation: A Statistical Analysis of Publicly Available Case Data. *J Clin Med* 2020;9(2) doi: 10.3390/jcm9020538 [published Online First: 2020/02/17]

5. Khalili M, Karamouzian M, Nasiri N, et al. Epidemiological Characteristics of COVID-19; a Systematic Review and Meta-Analysis. *Plos One* 2020;Submitted

6. Bi Q, Wu Y, Mei S, et al. Epidemiology and Transmission of COVID-19 in Shenzhen China: Analysis of 391 cases and 1,286 of their close contacts. *medRxiv* 2020 doi: 10.1101/2020.03.03.20028423

7. Mizumoto K, Kagaya K, Zarebski A, et al. Estimating the Asymptomatic Proportion of 2019 Novel Coronavirus onboard the Princess Cruises Ship, 2020. *medRxiv* 2020:2020.02.20.20025866. doi: 10.1101/2020.02.20.20025866

8. Day M. Covid-19: four fifths of cases are asymptomatic, China figures indicate. *BMJ* 2020;369:m1375. doi: 10.1136/bmj.m1375 [published Online First: 2020/04/02]

9. Coronavirus disease 2019 (COVID-19) pandemic: increased transmission in the EU/EEA and the UK – seventh update [Available from: <https://www.ecdc.europa.eu/sites/default/files/documents/RRA-seventh-update-Outbreak-of-coronavirus-disease-COVID-19.pdf>.

10. Vital Surveillances: The Epidemiological Characteristics of an Outbreak of 2019 Novel Coronavirus Diseases (COVID-19) — China, 2020 [Available from: <http://weekly.chinacdc.cn/en/article/id/e53946e2-c6c4-41e9-9a9b-fea8db1a8f51>.

11. Hauser A, Counotte MJ, Margossian CC, et al. Estimation of SARS-CoV-2 mortality during the early stages of an epidemic: a modelling study in Hubei, China and northern Italy. *medRxiv* 2020:2020.03.04.20031104. doi: 10.1101/2020.03.04.20031104

12. Verity R, Okell LC, Dorigatti I, et al. Estimates of the severity of coronavirus disease 2019: a model-based analysis. *Lancet Infect Dis* 2020 doi: 10.1016/s1473-3099(20)30243-7 [published Online First: 2020/04/03]

13. Coronavirus: summary of persons being monitored, persons under investigation, and cases [Available from: <https://floridadisaster.org/globalassets/covid19/dailies/covid-19-data---daily-report-2020-04-25-0956.pdf>.

14. Petrilli CM, Jones SA, Yang J, et al. Factors associated with hospitalization and critical illness among 4,103 patients with COVID-19 disease in New York City. *medRxiv* 2020:2020.04.08.20057794. doi: 10.1101/2020.04.08.20057794

15. Richardson S, Hirsch JS, Narasimhan M, et al. Presenting Characteristics, Comorbidities, and Outcomes Among 5700 Patients Hospitalized With COVID-19 in the New York City Area. *JAMA* 2020 doi: 10.1001/jama.2020.6775 [published Online First: 2020/04/22]
