# Supplementary material for: Impact of alternative Non-Pharmaceutical Interventions strategies for controlling COVID-19 outbreak in Bangladesh: A modeling study
Source: PLoS One. 2024 Feb 23;19(2):e0293863. doi: 10.1371/journal.pone.0293863 (PMC10890722; doi:10.1371/journal.pone.0293863)
Supplement: Attachment — Submitted filename: CoMo_Bangladesh_Schooling_Response_to_Reviewers.docx [file pone.0293863.s003.docx]

March 18, 2021

Kin On Kwok, Ph.D
Academic Editor
PLOS ONE

Dear Dr Kwok:

Thank you very much for your inquiry and feedback from the reviewers of our article” Impact of alternative Non-Pharmaceutical Interventions strategies for controlling COVID-19 outbreak in Bangladesh: a modelling study”.

Following your guidance, ee have made the following corrections and changes:

- Added more references and context for the CoMo model cited in the paper
- Ensured text of paper refers to Tables 1-3 at end of paper.
- Provided more detail about the COVID-19 related death rate in Bangladesh cited in the paper.
- Made substantial editions to the text to improve the language and flow.

We also attach excel files of Tables 1-4 for your reference; these are input data that populate the CoMo model.

We hope the changes meet the requirements needed to be accepted for publication. We are of course happy to make any additional needed changes.

Once again thank you very much for your consideration and hope to hear fro you soon.

Best regards.

Shafiun Shimul
