# Supplementary material for: Impact of alternative Non-Pharmaceutical Interventions strategies for controlling COVID-19 outbreak in Bangladesh: A modeling study
Source: PLoS One. 2024 Feb 23;19(2):e0293863. doi: 10.1371/journal.pone.0293863 (PMC10890722; doi:10.1371/journal.pone.0293863)
Supplement: Attachment — Submitted filename: Response to the reviewer.docx [file pone.0293863.s004.docx]

**Response to the reviewer’s comments**

**Manuscript: Impact of alternative Non-Pharmaceutical Interventions strategies for controlling COVID-19 outbreak in Bangladesh: a modeling study**

Reviewer #1

Still, there are plenty of language-related errors. I recommend authors use proofreading tools such as Grammarly to check the language errors.

Response: the manuscript is thoroughly revised and proofread as suggested by the author. Edited by a native English author and by Grammarly.

In the Data section, line 182 is an incomplete sentence. Several such mistakes can be found in the document.

Response: corrected

Although the authors have explained the CoMo model, it is not sufficient. Please include a technical explanation of the model (mathematical explanation).

Response: added the core mathematical equations
