# Supplementary material for: Impact of alternative Non-Pharmaceutical Interventions strategies for controlling COVID-19 outbreak in Bangladesh: A modeling study
Source: PLoS One. 2024 Feb 23;19(2):e0293863. doi: 10.1371/journal.pone.0293863 (PMC10890722; doi:10.1371/journal.pone.0293863)
Supplement: Attachment — Submitted filename: Editors suggestions and our respones.docx [file pone.0293863.s005.docx]

**Editor’s suggestions and our response:**

a) get their written agreement to share the model details (unless it was publicly available) and upload that as a supplementary file,

Response:

We have revised the manuscript describing how to replicate the results. We also provided the steps to replication using a publicly available source (page 7, lines 179-183). Details were provided in the supplement. Supplements have been uploaded.

b) reference all the relevant literature sources used for calibrating the model's parameters,

Response: sources of information were included in the supplement (Table A1).

and c) clarify the relative contributions of yours relative to the Oxford team in the manuscript (this was not very clear until now, even after I read the paper twice).

Response:

We basically used a CoMo model developed by CoMo team. A footnote (footnote 2, page 6) has been added to clarify this matter.
